# Development of a novel spatiotemporal depletion system for cellular cholesterol

**DOI:** 10.1016/j.jlr.2022.100178

**Published:** 2022-02-08

**Authors:** Ha Pham, Indira Singaram, Jiachen Sun, Arthur Ralko, Madalyn Puckett, Ashutosh Sharma, Alice Vrielink, Wonhwa Cho

**Affiliations:** 1 Department of Chemistry, University of Illinois at Chicago, Chicago, IL, USA; 2 School of Molecular Sciences, University of Western Australia, Perth, Western Australia, Australia

**Keywords:** cholesterol, site-specific depletion, cholesterol quantification, cholesterol signaling, plasma membrane, lysosomes, chemically induced dimerization, FRB, FKBP-rapamycin binding, IPM, inner leaflet of the PM, LUVs, large unilamellar vesicles, MβCD, methyl-β-cyclodextrin, mTORC1, mechanistic target of rapamycin complex1, NPC1, Niemann-Pick C1, OPM, outer leaflet of the PM, PM, plasma membrane, POPS, 1-palmitoyl-2-oleoyl-sn-glycero-3-phosphoserine, pS6K, phospho-p70 S6K, S6K, S6 kinase, SPR, surface plasmon resonance, TSC2, tuberous sclerosis complex 2

## Abstract

Cholesterol is an essential component of mammalian cell membranes whose subcellular concentration and function are tightly regulated by de novo biosynthesis, transport, and storage. Although recent reports have suggested diverse functions of cellular cholesterol in different subcellular membranes, systematic investigation of its site-specific roles has been hampered by the lack of a methodology for spatiotemporal manipulation of cellular cholesterol levels. Here, we report the development of a new cholesterol depletion system that allows for spatiotemporal manipulation of intracellular cholesterol levels. This system utilizes a genetically encoded cholesterol oxidase whose intrinsic membrane binding activity is engineered in such a way that its membrane targeting can be controlled in a spatiotemporally specific manner via chemically induced dimerization. In combination with in situ quantitative imaging of cholesterol and signaling activity measurements, this system allows for unambiguous determination of site-specific functions of cholesterol in different membranes, including the plasma membrane and the lysosomal membrane.

Cholesterol is a major and essential component of the mammalian cell membranes (1, 2, 3, 4). Although cholesterol biosynthesis takes place in the endoplasmic reticulum, a large majority of unesterified cholesterol is found in the plasma membrane (PM) where it controls mechanical properties of the lipid bilayer (1, 2), including rigidity and permeability (5). Cholesterol has been also implicated in cell signaling through the formation of membrane microdomains at the PM, including lipid rafts (6, 7, 8), which may compartmentalize diverse cell signaling events (9, 10, 11, 12). Recently, cholesterol has been reported to directly interact with a wide variety of cellular proteins (13). Multiple studies have reported that cholesterol regulates the structure and function of various integral membrane proteins at the PM (14), such as ion channels (15, 16) and G protein-coupled receptors (17) through direct interaction. Accumulating evidence also supports the notion that cholesterol in the inner leaflet of the PM (IPM) regulates cytosolic signaling proteins through direct and specific interactions (18, 19, 20, 21). Furthermore, it has been reported that cholesterol mediates cell signaling events at lysosomes by activating the mechanistic target of rapamycin complex1 (mTORC1) via an SLC38A9-Niemann-Pick C1 (NPC1) signaling complex (22). Together, these reports underscore the need to develop a robust method to precisely control the cholesterol level in a site-specific manner to determine cellular functions of cholesterol. The cellular cholesterol levels have been commonly manipulated by two methods: chemical extraction and enrichment of cholesterol by methyl-β-cyclodextrin (MβCD) and MβCD-cholesterol adducts, respectively (23) and inhibition of cellular de novo cholesterol biosynthesis by statins (24). Despite their popularity due to experimental convenience, these methods offer no spatiotemporal control and cause nonspecific cholesterol depletion that could exert major deleterious effects on cell physiology.

To overcome these obstacles, we have developed a new inducible, site-specific cholesterol depletion system. The system employs a genetically encoded bacterial cholesterol oxidase whose membrane binding activity is altered in such a way that allows spatiotemporally specific control of its membrane targeting by chemically induced dimerization with a partner protein located in a specific membrane site. In combination with in situ quantitative imaging of cholesterol (25, 26, 27) and signaling activity assays, this system allows for unambiguous determination of site-specific functions of cholesterol in the cytofacial leaflets of diverse cell membranes, including PM and the lysosomal membrane.

## Materials and methods

### Materials

Cholesterol, POPC, and 1-palmitoyl-2-oleoyl-*sn*-glycero-3-phosphoserine (POPS) were purchased from Avanti Polar Lipids. Human Wnt3a protein was purchased from R&D Systems (Cat no. 5036-WN-010; lot no. RSK9221052). siRNA for human TSC2 was purchased from Integrated DNA Technologies (Ref. no. 311363879) and the transfection reagent JetPRIME was from Polyplus-transfection. Antibodies against β-catenin (Cat no. 9587S), active (nonphospho) β-catenin (Cat no. 8814S), Tuberin/TSC2 (Cat no. 4308S), p70 S6 kinase (S6K) (Cat no. 2708S), phospho-p70 S6K (pS6K) (Cat no. 9205S), and glyceraldehyde 3-phosphate dehydrogenase (Cat no. 5174S) were purchase from Cell Signaling Technologies. Anti-Rabbit IgG, HRP-linked antibody (Cat no. 7074S) and anti-Mouse IgG as well as HRP-linked antibody (Cat no. 7076S) were also from Cell Signaling Technologies. HEK293T and HeLa cells were purchased from ATCC. Chemicals for cholesterol oxidase assay activity, including 4-aminoantipyrine (Cat no. A4382-10G), phenol (Cat no. P1037-25G), and peroxidase from horseradish (Cat no. P8375-1KU) were purchased from Millipore Sigma.

### Bacterial expression and purification of cholesterol oxidase

All *st*CholOx proteins were produced using the pET-30a vector with an N-terminal His_6_-tag. *Escherichia coli* BL21 RIL codon plus cells (Stratagene) were transformed with the construct for bacterial protein expression. A preculture solution was prepared from a single colony in 10 ml of LB media with 50 μg/ml kanamycin and incubated in a shaker at 37°C overnight or until the media became cloudy. A 5 ml aliquot of the preculture was transferred to 500 ml of the main culture medium containing 50 μg/ml kanamycin, and the mixture was incubated in a shaker at 37°C until the absorbance at 600 nm reached 0.6. Protein expression was then induced at 18°C with 0.5 mM isopropyl β-d-1-thiogalactopyranoside for 16 h. The cells were collected by centrifugation of the culture medium at 4,000 *g* for 10 min. Cell pellets were stored at −80°C until use. The cells were resuspended with 20 ml of the lysis buffer (50 mM Tris-HCl, pH 7.9, with 300 mM NaCl, 10 mM imidazole, 0.1% Triton X-100, 1 mM phenylmethanesulfonylfluoride, and 1 mM dithiothreitol) and lysed by sonication. The lysate was centrifuged at 44,000 *g* for 30 min, the clear supernatant was mixed with 1 ml of Ni-NTA agarose resin (Marvelgent Biosciences Inc), and the mixture was incubated at 4°C for 2 h with gentle mixing. The resin was washed with consecutively 100 ml of the wash buffer 1 (50 mM Tris-HCl, pH 7.9, with 300 mM NaCl, and 20 mM imidazole), 50 ml of the wash buffer 2 (50 mM Tris-HCl, pH 7.9, with 500 mM NaCl, and 20 mM imidazole), and 100 ml of the wash buffer 3 (20 mM Tris-HCl, pH 7.9 with 160 mM NaCl, and 40 mM imidazole). The protein was then eluted from the resin with 1 ml of the elution buffer (50 mM Tris-HCl, pH 7.9, with 300 mM NaCl, and 300 mM imidazole). The protein concentration was determined by the Bradford assay.

### In vitro cholesterol oxidase activity assay

10 μl of purified cholesterol oxidase WT or mutant (400 μg/ml) was added to 200 μl of reaction buffer (20 mM Tris buffer containing 160 mM NaCl at pH 7.4%, 0.34% Triton X-100, 1.4 mM 4-aminoantipyrine, 21 mM phenol, 1 mM cholesterol, and 5 units/ml horseradish peroxidase) in a 96-well plate. The reaction was incubated at 37°C for 5 min. Absorbance at 500 nm in the assay was monitored with the BioTek Synergy™ Neo spectrofluorometer.

### Surface plasmon resonance analysis

All surface plasmon resonance (SPR) measurements were performed at 23°C in a degassed 20 mM Tris, pH 7.4, buffer solution containing 0.16 M NaCl using a lipid-coated L1 chip in the BIACORE X100 system (Cytiva) as described previously (28, 29). Large unilamellar vesicles (LUVs) with an average diameter of 100 nm were prepared by extrusion through a 100-nm diameter polycarbonate membrane (Avanti Polar Lipids) in an Avanti Mini-Extruder (Avanti Polar Lipids) as described previously (30). POPC/POPS/cholesterol (60:20:20) and POPC/POPS (80:20) LUVs were used as the active surface and the control surface, respectively. Sensorgrams show the difference in SPR resonance values (in resonance unit) between the active and control surfaces as a function of time. SPR measurements were carried out at a flow rate of 30 μl/min to circumvent cholesterol degradation during binding measurements. Also, only the association phases were monitored and analyzed due to potential membrane deformation during the dissociation phases.

### Cell culture and confocal imaging of mTagBFP-*st*CholOx

HeLa and HEK293T cells were maintained in the Dulbecco’s Modified Eagle Medium (Gibco), supplemented with 10% fetal bovine serum (Sigma), 2 mM glutamine (Gibco), and 1% penicillin/streptomycin (Gibco) at 37°C with 5% CO_2_. The recombinant mTagBFP-*st*CholOx was subcloned into a pcDNA3 vector. Membrane binding-deficient mutants of *st*CholOx and their FK506 binding protein (FKBP)-tagged derivatives were then generated using a Q5® site-directed mutagenesis kit (NEB). The same number (2.5 × 10^4^) of HeLa or HEK293T cells were seeded at 70%–80% confluence on a glass bottom 35 mm cell culture dish. After 24 h, the pcDNA3 plasmid encoding mTagBFP-*st*CholOx (WT or derivatives) was transfected into the cells using the JetPRIME system (Polyplus-transfection) according to the manufacturer’s protocol. The following day, the growth medium was switched to Invitrogen™ Live Cell Imaging Solution (ThermoFisher) to minimize the fluorescence background. Fluorescence images of mTagBFP-*st*CholOx and their derivatives were obtained with the custom-designed six channel Olympus FV3000 confocal microscope with the environmentally controlled full enclosure incubator (CellVivo). The cells were maintained at 37°C and with 5% CO_2_ atmosphere throughout the imaging period to maintain cell viability. Collected images were imported to and analyzed in ImageJ or Image-Pro Plus 7.

### Quantitative cholesterol imaging

Spatiotemporally resolved in situ quantification of intracellular cholesterol in HeLa and HEK293T cells was performed using ratiometric cholesterol sensors, WCR-YDA (all intracellular cholesterol), and DAN-D4 (cholesterol in the outer leaflet of the PM, OPM), as described previously (25, 26). WCR-YDA and DAN-D4 were prepared and calibrated using giant unilamellar vesicles as described previously (25, 26). WCR-YDA was microinjected into HeLa or HEK293T cells, and the cholesterol concentration in the IPM and lysosomes was determined using in-house programs written in MATLAB as described (26). DAN-D4 was added to the cultured medium for cell surface cholesterol quantification. The three-dimensional display of local lipid concentration profile was calculated using the Surf function in MATLAB.

### Site-specific cholesterol depletion

For transient expression of Lyn-FRB and FKBP_2_-mTagBFP-WVR for PM targeting, Lyn-FRB and FKBP_2_-mTagBFP-WVR were separately subcloned into the pcDNA3 vector. Equal amounts of the resulting plasmids were transfected into HeLa or HEK293T cells using the JetPRIME system (Polyplus-transfection) according to the manufacturer’s protocol. The following day, the growth medium was switched to Invitrogen™ Live Cell Imaging Solution, and the PM targeting of FKBP_2_-mTagBFP-WVR was induced by adding rapalog to the solution to a final concentration of 50 nM. Lysosomal targeting of FKBP_2_-mTagBFP-WVR was performed by the same protocol except that Lamp1-iRFP713-FRB was used in lieu of Lyn-FRB. For stable expression of Lyn-FRB and FKBP_2_-mTagBFP-WVR, a dual-expression vector was prepared by subcloning the genes encoding FKBP_2_-mTagBFP-WVR under the TRE_tight_ promoter, for a tight-control expression of the gene by doxycycline, and Lyn-FRB under the CMV promoter into a PiggyBac vector using In-Fusion® Cloning Kit. The plasmid for the dual expression of Lamp1-iRFP713-FRB and FKBP_2_-mTagBFP-WVR for lysosomal targeting was prepared in the same manner except that pCMV-Lamp1-iRFP713-FRB was used in lieu of pCMV-Lyn-FRB. The dual-expression plasmid (1.5 μg) and the recombination helper plasmid pSPB-Transposase (0.6 μg) were transfected into 70%–80% confluent HeLa (or HEK293T) cells in a 6-well plate using the JetPRIME system (Polyplus-transfection) according to the manufacturer’s protocol. Untransfected cells were kept in a separate well as a control. After 24 h of transfection, the growth medium was replaced with the selection medium (Dulbecco's modified Eagle's medium with 10% fetal bovine serum, 200 μg/ml hygromycin, 1% penicillin and streptomycin). The medium was replaced every other day until the cells in the control well were completely dead. Successful stable expression of the PM or lysosomal depletion system was confirmed with the cellular mTagBFP (after doxycycline induction overnight) or iRFP713 signal by confocal microscopy. These stably transfected cells were maintained in the growth medium containing 100 μg/ml hygromycin. Expression of FKBP_2_-mTagBFP-WVR in these cells was induced by overnight incubation with 50 ng/ml doxycycline while Lyn-FRB or Lamp1-iRFP713-FRB was constitutively expressed. Cholesterol depletion in these cells was induced by 50 nM of rapalog and monitored by ratiometric cholesterol imaging.

### Cell signaling activity assays

HEK293T cells stably expressing the site-specific cholesterol depletion system were seeded onto a 6-well plate at 80% confluence. After overnight incubation with 50 ng/ml doxycycline to induce expression FKBP_2_-mTagBFP-WVR, the cells were treated with 50 ng/ml Wnt3a for 16 h. The cells were treated with 50 nM of rapalog for the indicated periods before the amounts of active (unphosphorylated) and total β-catenin were measured by Western blot analysis. For lysosome-specific cholesterol depletion, HEK293T cells stably expressing the site-specific cholesterol depletion system were seeded at 80% confluence in a 6-well plate. On the next day, the cells were transfected with 30 nM TSC2 siRNA. After 48 h, the cells were treated with 50 nM rapalog for the indicated periods and the amounts of pS6K and total S6K were measured by Western blot analysis.

### Western blot analysis

Treated cells were lysed in the cell lysis buffer (20 mM Tris-HCl, pH 7.5, containing 150 mM NaCl, 1 mM Na_2_EDTA, 1 mM EGTA, 1% Triton-X, protease inhibitors, and phosphatase inhibitors (1 mM Na_3_VO_4_, 1 mM NaF, 1 μg/ml leupeptin, 1 mM phenylmethanesulfonylfluoride, 1.5 mM benzamidine, and 2 μg/ml pepstatin)). The total protein concentration of the cell lysate was determined by the Pierce bicinchoninic acid protein assay kit (Thermo scientific). 30 μg of proteins were loaded onto a polyacrylamide gel to run sodium dodecyl sulfate polyacrylamide gel electrophoresis. The proteins were separated and transferred to a 0.45 μm polyvinylidene difluoride membrane (Thermo Fisher). The membrane was blocked with 5% bovine serum albumin for 1 h and incubated overnight at 4°C with a primary antibody (1:1,000 dilution for all antibodies). After the unbound antibodies were removed by washing with 0.1% Tris buffer saline with 0.1% Tween20, the membranes were incubated with the HRP-linked secondary antibody (1:5,000 dilution) for 1 h at room temperature. The membranes were washed three more times with 0.1% Tris buffer saline with 0.1% Tween20 to remove the unbound HRP-linked secondary antibody before imaging. The intensity of protein bands in the gel was visualized using the Enhanced chemiluminescence reagents (Thermo Fisher) and documented by the Azure 500Q Imaging System.

### Quantification and statistical analysis

Statistical significance was calculated by the Student's *t* test. The number of experiments, the number of total cells analyzed (n), and significance are reported in the figure legends. Sample sizes for cellular imaging and assays were chosen as the minimum number of independent observations required for statistically significant results.

## Results

### Engineering of cholesterol oxidase

Cholesterol oxidases are secreted bacterial enzymes that catalyze the first step in the degradation of cholesterol (31). These flavin adenine dinucleotide-containing enzymes catalyze two-step chemical conversions of cholesterol to cholest-4-en-3-one. Cholesterol oxidases are constitutively active enzymes that spontaneously bind cholesterol-containing membranes and initiates the first step in cholesterol degradation (31). Cholesterol oxidases have thus been commonly used for determination of serum cholesterol levels (31) and for depleting cholesterol from the OPM of mammalian cells for functional studies (31). Recently, an engineered split cholesterol oxidase was introduced as an inducible intracellular agent, but its slow mode of action and lack of spatial control have limited its application (32).

**Fig. 1:**
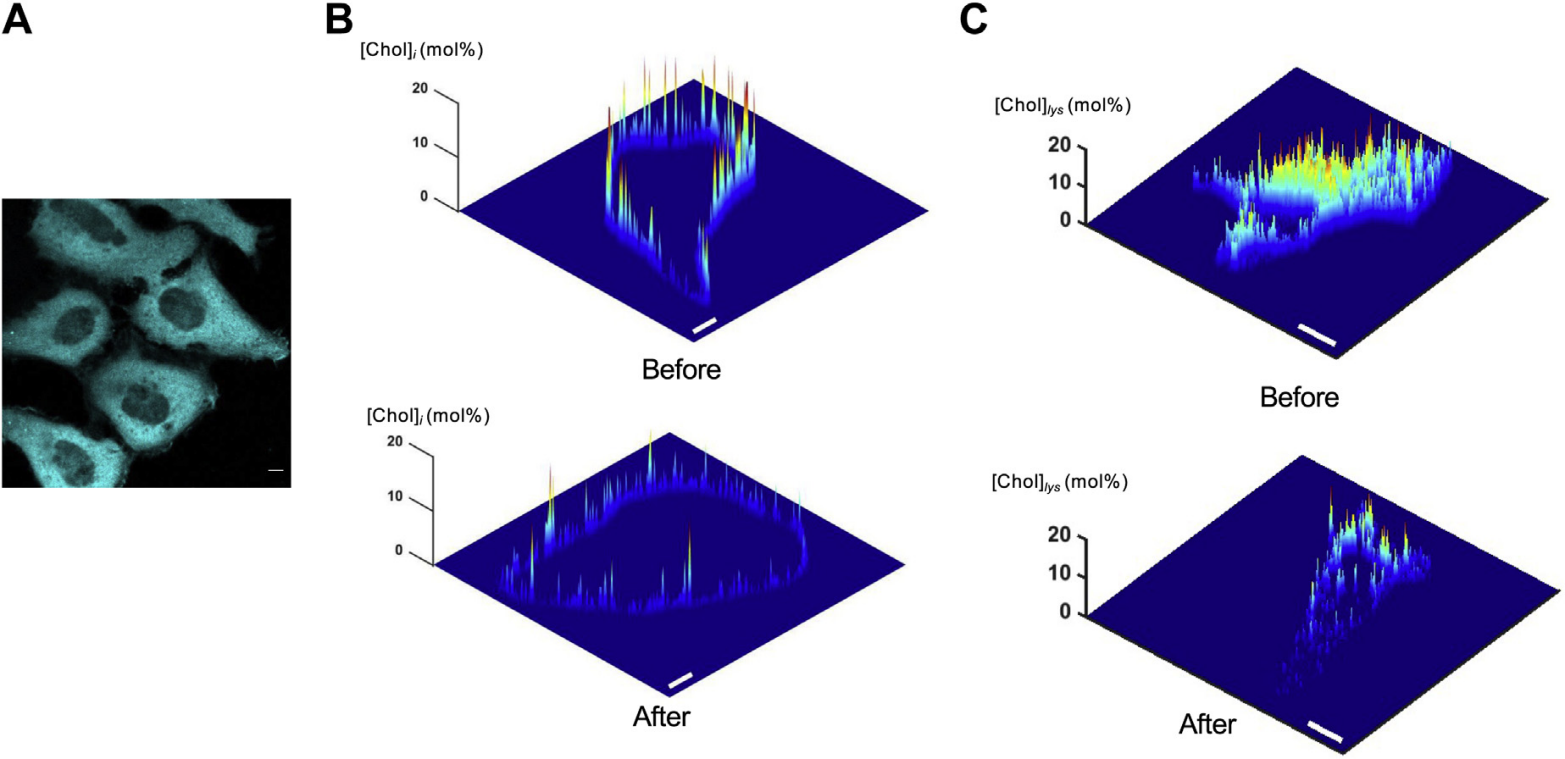
Effects of exogenous expression of *Streptomyces* sp. cholesterol oxidase (*st*CholOx) on cellular cholesterol levels. A: Cellular distribution of mTagBFP-*st*CholOx. B, C: Spatially resolved concentration profiles of cholesterol at IPM ([Chol]_*i*_) (B) and at the cytofacial leaflet of the lysosomal membrane ([Chol]_*lys*_) (C) calculated from two-channel cross-sectional images of HeLa cells microinjected with the ratiometric cholesterol sensor, WCR-YDA, at a given time. Each image is a representative of more than 10 cells analyzed (n > 10). Spatially averaged [Chol]_*i*_ values before and after mTagBFP-*st*CholOx expression are 3.5 ± 0.2 and 0.3 ± 0.15 mol%, respectively. Spatially averaged [Chol]_*lys*_ values before and after mTagBFP-*st*CholOx expression are 9.0 ± 0.5 and 1.5 ± 0.4 mol%, respectively. The *z*-axis scale indicates [Chol]_*i*_ and [Chol]_*lys*_ in mol%. A pseudo-coloring scheme with red and blue representing the highest (20 mol%) and the lowest (0 mol%) concentration, respectively, is used to illustrate the spatial [Chol]_*i*_ and [Chol]_*lys*_ heterogeneity. The images shown here are representatives of all collected data. n = 5 (A), 15 (B), and 12 (C). The scale bars indicate 10 μm. IPM, inner leaflet of the PM.

We first exogenously expressed cholesterol oxidase from *Streptomyces* sp. (strain SA-COO) (will be referred to as *st*CholOx hereafter) as a monomeric blue fluorescent protein (mTagBFP)-tagged protein in HeLa cells (Fig. 1A). Although the protein was primarily located in the cytosol (Fig. 1A), our quantitative cholesterol imaging using WCR-YDA (25, 26) showed that mTagBFP-*st*CholOx reduced the cholesterol concentration at the IPM ([Chol]_*i*_) from 3.5 to 0.3 mol% (Fig. 1B) and the cholesterol concentration at the cytofacial face of lysosomes ([Chol]_*lys*_) from 9.0 to 1.5 mol% (Fig. 1C). This confirms that *st*CholOx is a constitutively active enzyme that causes nonspecific cholesterol depletion as do MβCD and statins if overexpressed without spatiotemporal control in mammalian cells.

**Fig. 2:**
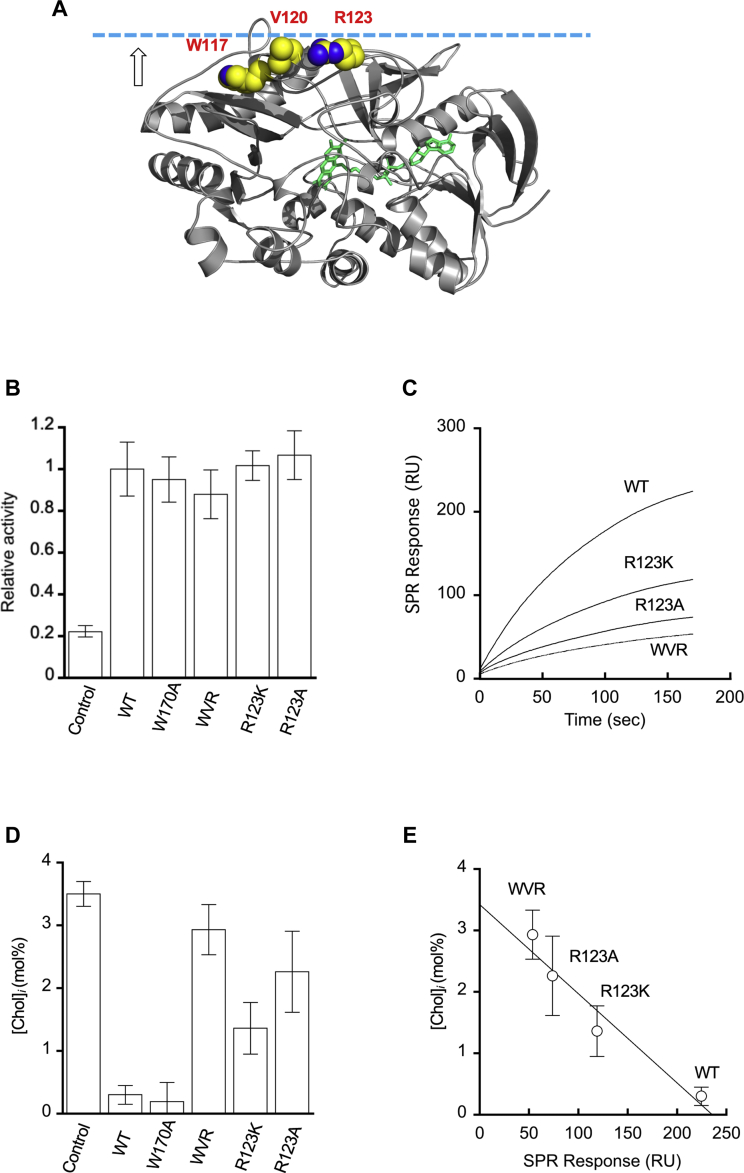
Catalytic and membrane binding activity of *st*CholOx WT and mutants. A: The crystal structure of *st*CholOx (protein data bank ID: 1B4V) shown in a ribbon diagram. The flavin adenine dinucleotide cofactor in the active site is highlighted in green. The residues potentially involved in membrane binding are shown as van der Waals spheres and labeled. The molecule is oriented with its putative membrane binding surface pointing upward (arrow). The dotted cyan line illustrates the hypothetical membrane surface. B: Relative enzyme activity of purified *st*CholOx WT and mutants by the cholesterol oxidase activity assay in mixed micelles. The control measurement was performed without cholesterol oxidase. C: Relative affinity of *st*CholOx WT and mutants for POPC/POPS/cholesterol (70:20:10 in mole ratio) vesicles determined by SPR analysis. The protein concentration was 100 nM. Each sensorgram is a representative of three independent measurements. D: The effect of *st*CholOx WT and mutants on [Chol]_*i*_ in HeLa cells expressing mTagBFP-*st*CholOx WT or mutants measured by microinjected WCR-YDA. The bar graphs show average ± S.D. values (n = 3 for B and n = 8–10 cells for D). E: Correlation between the relative membrane affinity of *st*CholOx WT and mutants and their effects on [Chol]_*i*_. [Chol]_*i*_ in HeLa cells expressing *st*CholOx proteins is inversely correlated with their membrane affinity. POPS, 1-palmitoyl-2-oleoyl-sn-glycero-3-phosphoserine; SPR, surface plasmon resonance.

To convert *st*CholOx into a spatiotemporally inducible cholesterol depletion agent, we engineered *st*CholOx in such a manner that its interfacial catalysis may be controlled in a spatiotemporally specific manner. Interfacial catalysis of enzymes that act on membrane lipids, such as lipases and cholesterol oxidases, involves two separate processes, that is, initial membrane binding of the protein and subsequent and/or concomitant catalytic steps (33, 34). Our previous studies on numerous lipid binding domains and proteins have shown that their membrane binding surfaces are composed of a cluster of basic, aromatic, and hydrophobic residues surrounding a lipid binding pocket (28, 35, 36). Electrostatic potential calculation and surface cavity analysis (19, 20, 28, 36) of a known crystal structure of *st*CholOx (37) predicted its putative membrane surface (Fig. 2A). Based on this prediction, we designed mutations to systematically lower the membrane binding activity of *st*CholOx. Specifically, we made several single- or multi-site mutations of those residues on the putative membrane surface, including W170A, R123K, R123A, and W117A/V120A/R123A (will be referred to as WVR) and measured their effects on the interfacial binding and the catalytic process separately.

First, we bacterially expressed *st*CholOx WT and mutants and measured their relative enzyme activity by a reported cholesterol oxidase activity assay in mixed micelles (38, 39). Due to its hydrophobicity, cholesterol is buried within the hydrophobic core of the lipid bilayers, including cell membranes (40, 41). Thus, soluble proteins, such as cholesterol oxidase, have limited access to cholesterol (40, 41), and their membrane binding requires partial penetration into the hydrophobic core of the bilayers (26, 35). In general, lipids in mixed micelles are highly accessible due to low packing density (42) and thus detergent-dispersed cholesterol molecules should be much more easily accessible to *st*CholOx than those buried in the lipid bilayer. Thus, the enzymatic activity measured in this system largely reflects the catalytic efficiency of the enzyme. As shown in Fig. 2B, *st*CholOx and all mutants showed statistically indistinguishable cholesterol oxidase activity (i.e., *P* > 0.05), showing that our mutations did not significantly alter the catalytic efficiency of *st*CholOx and that all our mutants are catalytically competent. To assess the effect of mutations on membrane binding of *st*CholOx, we then measured their binding to LUVs composed of POPC/POPS/cholesterol (60:20:20 in mole ratio) coated on the sensor chip by SPR analysis. To minimize cholesterol modification during the binding measurement, we employed fast flow rate and the O_2_-free buffer solution. SPR sensorgrams of *st*CholOx WT and mutants show that the mutations reduced the affinity of the protein for cholesterol-containing vesicles to varying degrees, with the WVR mutation having the largest effect (Fig. 2C). We then exogenously expressed *st*CholOx WT and mutants in HeLa cells and measured their effects on [Chol]_*i*_ (Fig. 2D). Clearly, those mutants with lower membrane affinity had smaller effects on [Chol]_*i*_, with WVR causing the smallest decrease in [Chol]. As shown in Fig. 2E, the relative [Chol]_*i*_ values in HeLa cells expressing *st*CholOx WT and mutants are inversely correlated with their relative membrane binding affinity. Since WVR has the lowest basal activity to degrade cellular cholesterol due to its severely compromised membrane binding activity, we selected WVR for further engineering to generate an inducible cholesterol-depletion agent.

### Site-specific targeting of engineered cholesterol oxidase to IPM and lysosomes

**Fig. 3:**
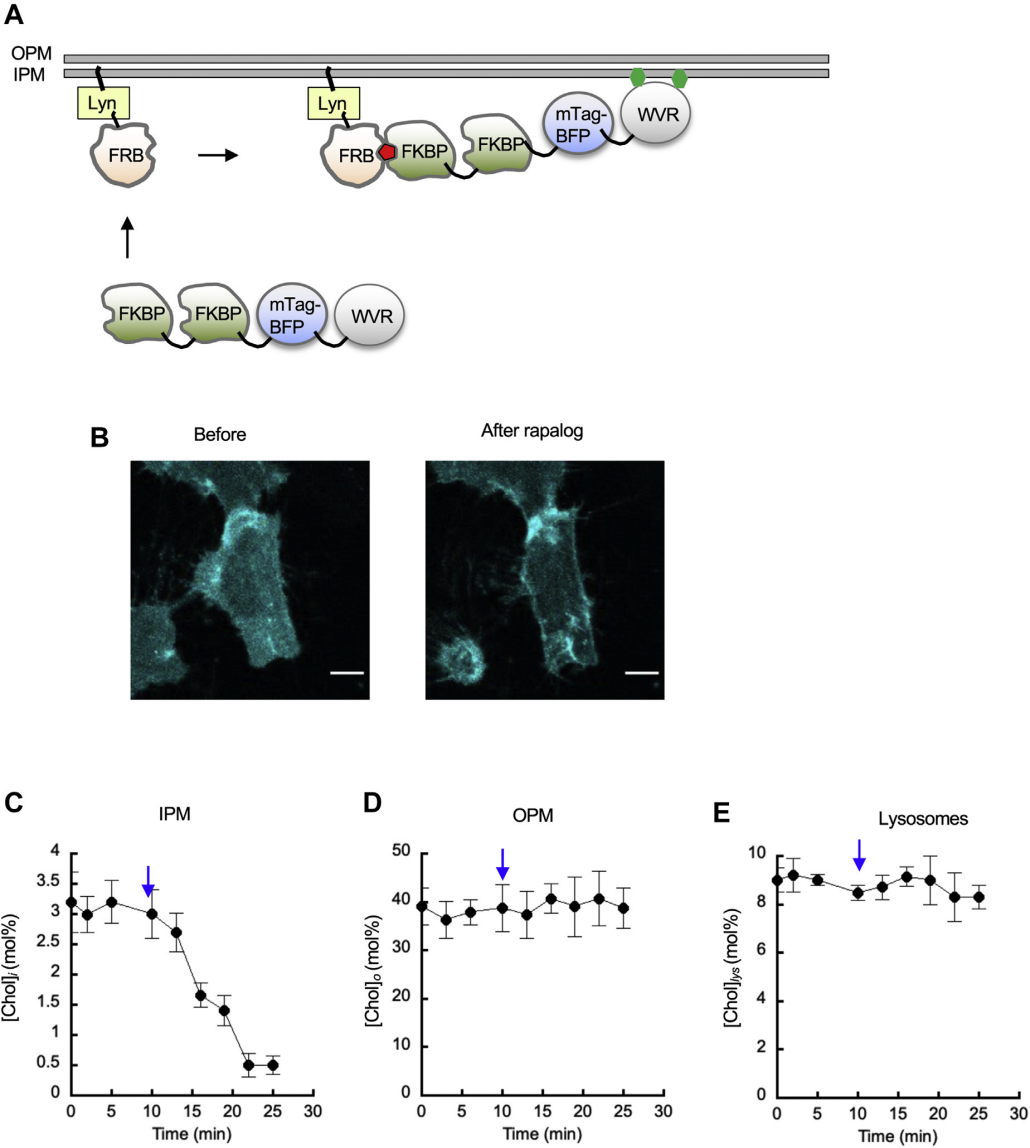
Design and characterization of the IPM-specific cholesterol depletion system. A: Targeting strategy. The best FKBP-FRB combination for IPM targeting of WVR was FKBP_2_-mTagBFP-WVR and Lyn-FRB. FKBP_2_-mTagBFP-WVR is primarily localized in the cytosol because of the comprised membrane binding affinity of WVR. Upon rapalog (red pentagon) stimulation, FKBP_2_-mTagBFP-WVR is specifically targeted to the IPM where Lyn-FRB is anchored via the N-terminal myristoyl group. WVR then depletes IPM cholesterol (green hexagon) by oxidation and isomerization. B: IPM targeting of FKBP_2_-mTagBFP-WVR in response to 50 nM rapalog in HeLa cells transiently expressing FKBP_2_-mTagBFP-WVR and Lyn-FRB. The images shown here are representative of 10 similar images. C–E: The time courses of cholesterol concentration at IPM ([Chol]_*i*_) (C), OPM ([Chol]_*o*_) (D), and lysosomes ([Chol]_*lys*_) (E) in HeLa cells transiently expressing FKBP_2_-mTagBFP-WVR and Lyn-FRB. Blue arrows indicate the timing of rapalog addition. The cells were treated with 50 nM rapalog for 15 min. Data points represent average ± S.D. values (n = 5–10). The scale bars indicate 10 μm. FRB, FKBP-rapamycin binding; IPM, inner leaflet of the PM; OPM, outer leaflet of the PM.

We then developed inducible site-specific cholesterol oxidase targeting systems by means of rapamycin-induced heterodimerization of FKBP and FKBP-rapamycin binding (FRB) domain of mTOR kinase (43, 44). Among the various combinations we tested, we found that the best FKBP-FRB combination for IPM targeting of WVR was mTagBFP-WVR with the tandem N-terminal FKBP tags (i.e., FKBP_2_-mTagBFP-WVR) and Lyn-FRB that is anchored to the PM due to the N-myristoylated sequence of Lyn (44) (Fig. 3A). As is the case with mTagBFP-*st*CholOx WT (see Fig. 1A), FKBP_2_-mTagBFP-WVR was relatively evenly distributed in the cytosol in unstimulated HeLa cells (Fig. 3B). Upon stimulation by rapalog, FKBP_2_-mTagBFP-WVR rapidly translocated to the PM (Fig. 3B). Although rapamycin yielded the same results, we exclusively used rapalog in this study to circumvent the inhibitory effect of rapamycin on mTORC1-mediated cell signaling. The kinetics of translocation is primarily limited by the diffusion and cell permeation of rapalog (43, 44). When [Chol]_*i*_ was quantified after rapalog stimulation, [Chol]_*i*_ was reduced to <0.5 mol% within 15 min (Fig. 3C). Neither localization of FKBP_2_-mTagBFP-WVR to other intracellular membranes (Fig. 3B) nor reduction in cholesterol concentration in the OPM ([Chol]_*o*_) (Fig. 3D) or other membranes (e.g., lysosomes ([Chol]_*lys*_)) (Fig. 3E) was observed, demonstrating that this system serves as an excellent tool to deplete IPM cholesterol in a spatiotemporally specific manner.

**Fig. 4:**
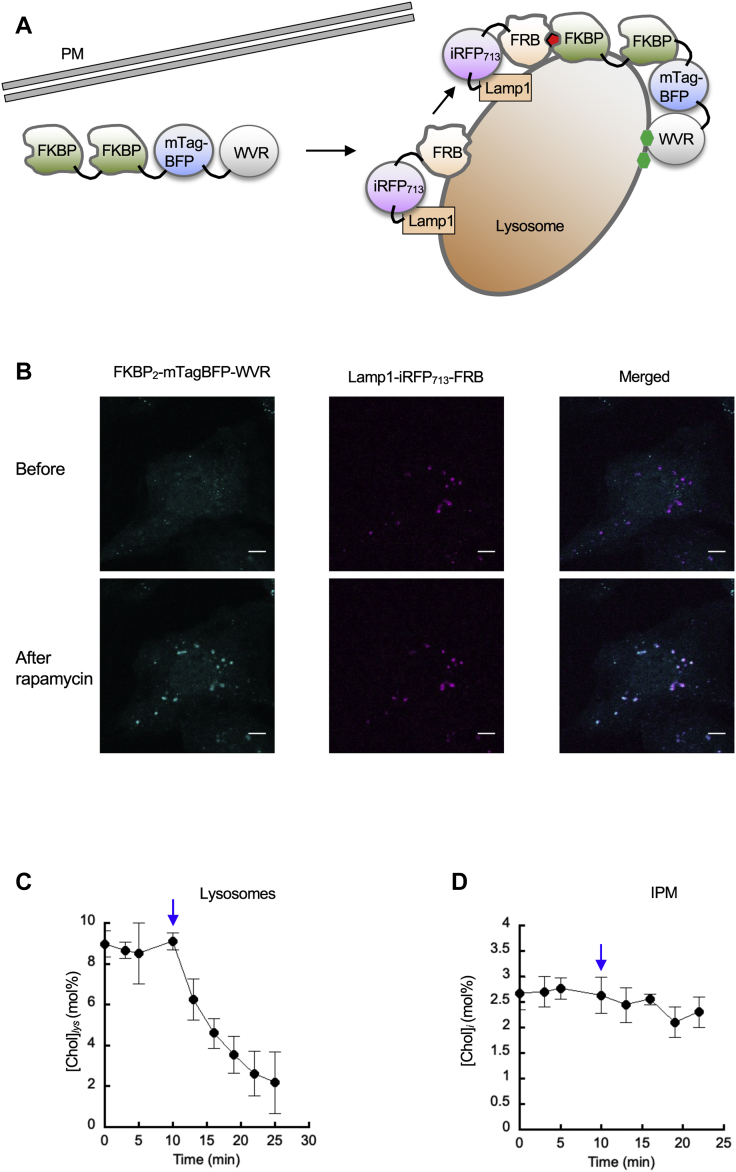
Design and characterization of the lysosome-specific cholesterol depletion system. A: The best FKBP-FRB combination for lysosome targeting of WVR was FKBP_2_-mTagBFP-WVR and Lamp1-iRFP_713_-FRB. See Fig. 3A for membrane targeting strategy. B: Lysosomal targeting of FKBP_2_-mTagBFP-WVR in response to 50 nM rapalog in HeLa cells transiently expressing FKBP_2_-mTagBFP-WVR and Lamp1-iRFP_713_-FRB. C, D: The time courses of [Chol]_*lys*_ (C) and [Chol]_*i*_ (D) in HeLa cells transiently expressing FKBP_2_-mTagBFP-WVR and Lamp1-iRFP_713_-FRB. Blue arrows indicate the timing of rapalog addition. The cells were treated with 50 nM rapalog for 15 min. Data points represent average ± S.D. values 5–10 cells. The scale bars indicate 10 μm. FRB, FKBP-rapamycin binding.

For lysosomal targeting of FKBP_2_-mTagBFP-WVR, we employed LAMP1-near-infrared fluorescent protein (iRFP_713_)-FRB that is exclusively localized to lysosomes (Fig. 4A) (44). Upon stimulation with rapalog, FKBP_2_-mTagBFP-WVR rapidly translocated to lysosomes (Fig. 4B). Translocation of FKBP_2_-mTagBFP-WVR to other cell membranes was minimal. Also, rapalog stimulation decreased [Chol]_*lys*_ from 9 to <2 mol% within 15 min (Fig. 4C). [Chol]_*i*_ was unaltered under these conditions (Fig. 4D), again underscoring the spatiotemporally specific nature of this cholesterol depletion system.

### Site-specific functions of cellular cholesterol probed with site-specific depletion systems

**Fig. 5:**
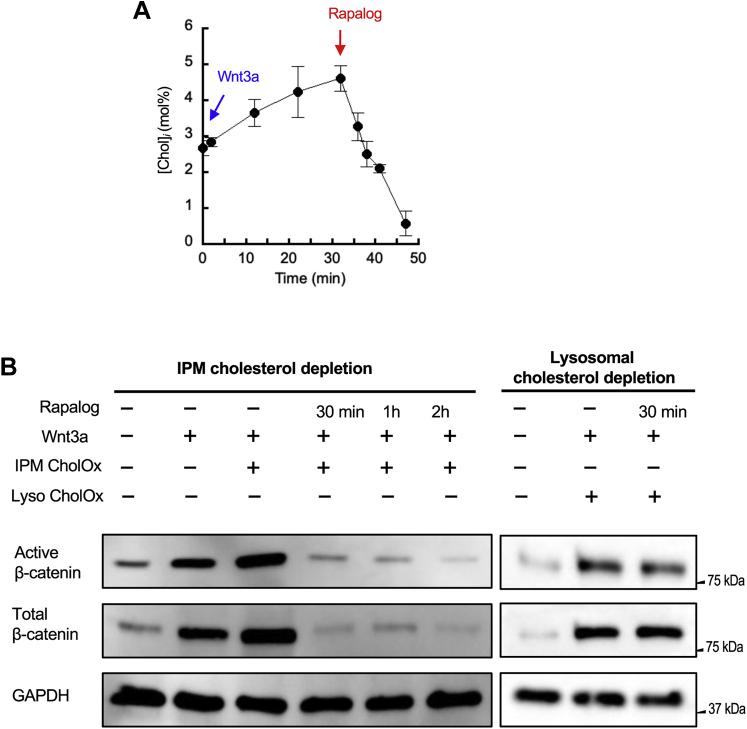
Site-specific function of IPM cholesterol determined by the site-specific cholesterol depletion system. A: Spatially averaged [Chol]_*i*_ in HEK293T cells stably expressing the IPM-specific cholesterol depletion system was plotted as a function of time after the addition of 50 ng/ml of Wnt3a and subsequently rapalog. B: Active and total β-catenin levels in WT HEK293T cells and HEK293T cells stably expressing the IPM-specific cholesterol depletion system (IPM CholOx) were monitored after the indicated treatments. As a control, we performed the same experiments using HEK293T cells stably expressing the lysosome-specific cholesterol depletion system (Lyso CholOx). 50 ng/ml of Wnt3a was incubated with the cells for 6 h to stimulate the Wnt-β-catenin signaling, and 50 nM rapalog (for 30 min, 1 h, or 2 h) was used to trigger cholesterol depletion. Glyceraldehyde 3-phosphate dehydrogenase (GAPDH) was used as a gel loading control. IPM, inner leaflet of the PM.

We previously reported that stimulation of HEK293T cells with Wnt3a induced the increase in [Chol]_*i*_ that leads to IPM recruitment of Dvl2 in a cholesterol-dependent manner, leading to the activation of the β-catenin-dependent canonical Wnt signaling pathway (19, 20, 21, 25). We tested if IPM cholesterol is specifically involved in this process using our IPM cholesterol-specific depletion system. For these functional measurements, we prepared HEK293T cells stably expressing Lyn-FRB and FKBP_2_-mTagBFP-WVR for experimental consistency. Consistent with the previous report (25), 50 ng/ml Wnt3a caused a continuous increase of [Chol]_*i*_ in these HEK293T cells, raising it from 2.5 to about 5.0 mol% in 30 min (Fig. 5A). Rapalog induction, however, rapidly reversed the course and reduced [Chol]_*i*_ to an undetectable level within 20 min (Fig. 5A). We then determined the effect of the change in [Chol]_*i*_ on the canonical Wnt signaling activity by measuring the stability of active (i.e., unphosphorylated) β-catenin and total β-catenin. After Wnt3a stimulation, the cellular level of active and total β-catenin, as measured by Western blot analysis, dramatically increased (Fig. 5B). Most importantly, site-specific depletion of IPM cholesterol by our system completely eliminated active and total β-catenin, whereas site-specific depletion of lysosomal cholesterol had no effect (Fig. 5B). These results confirm that IPM cholesterol is directly involved in canonical Wnt-β-catenin signaling.

**Fig. 6:**
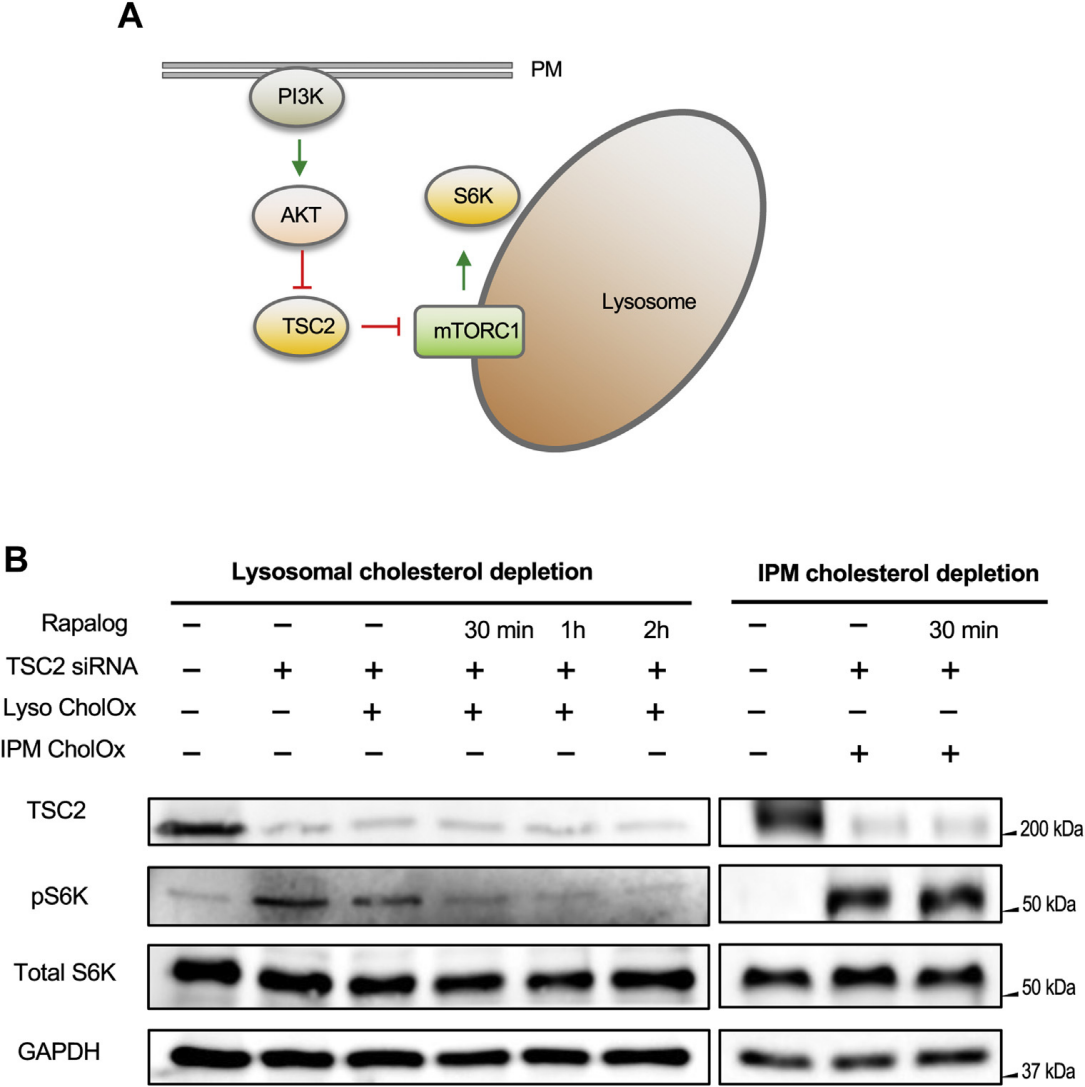
Site-specific function of lysosomal cholesterol determined by the site-specific cholesterol depletion system. A: mTORC1 signaling cascade. Upon cell stimulation (e.g., by growth factors), PI3K is activated, leading to Akt activation. Activated Akt then inhibits TSC2, which in turn activates mTORC1 by relieving its inhibition by TSC2. Thus, suppression of TSC2 expression by siRNA constitutively activates mTORC1 and its downstream protein, S6K. B: Phosphorylation of S6K in WT HEK293T cells and HEK293T cells stably expressing the lysosome-specific cholesterol depletion system (Lyso CholOx) was monitored after the indicated treatments. As a control, we performed the same experiments using HEK293T cells stably expressing the IPM-specific cholesterol depletion system (IPM CholOx). Expression of endogenous TSC2 was suppressed by 50 ng/ml siRNA, and 50 nM rapalog (for 30 min, 1 h, or 2 h) was used to trigger cholesterol depletion. GAPDH was used as a gel loading control. IPM, inner leaflet of the PM; mTORC1, mechanistic target of rapamycin complex1; PI3K, phosphoinositide 3-kinase; S6K, S6 kinase; TSC2, tuberous sclerosis complex 2.

We also measured the effect of reducing [Chol]_*lys*_ on the mTORC1 activity. mTORC1 is a serine/threonine-protein kinase complex that coordinates cellular growth and metabolism with environmental cues, including nutrients and growth factors (45). mTORC1 has been a subject of intense investigation because aberrant mTORC1 signaling is associated with cancers and various human diseases and because TORC1 also plays a key role in aging and lifespan (45). Upon cell stimulation by growth factors, Class I phosphoinositide 3-kinase is activated, leading to Akt activation (46, 47). Activated Akt then phosphorylates and inhibits tuberous sclerosis complex 2 (TSC2), which in turn activates mTORC1 by relieving the inhibition of mTORC1 by TSC2 (46, 47) (see Fig. 6A). p70 S6K is one of the many downstream effectors of mTORC1 and its phosphorylation at T389 (i.e., pS6K) is often used as a cellular readout of mTORC1 activity (22). Lysosomes have been long known as terminal degradation stations but have emerged as a cell signaling hub that governs cell growth, division, and differentiation more recently (48, 49). In particular, it has been reported that cholesterol at lysosomes activates mTORC1 via an SLC38A9-NPC1 signaling complex (22). It is not known, however, whether cholesterol in the cytofacial or luminal leaflets of lysosomal membranes is involved in this process. We thus used our lysosomal-specific depletion system to address this question. For this experiment, we prepared HEK293T cells stably expressing Lamp1-FRB and FKBP_2_-mTagBFP-WVR in which expression of endogenous TSC2 was suppressed by siRNA (Fig. 6B). As reported previously (22), knockdown of TSC2 results in constitutive activation of mTORC1 and S6K, as indicated by constitutive phosphorylation of S6K (Fig. 6B). When lysosome targeting of FKBP_2_-mTagBFP-WVR was triggered by rapalog, the pS6K band was eliminated within 30 min (Fig. 6B). In contrast, site-specific depletion of IPM cholesterol has no effect on S6K phosphorylation under the same conditions. These results show that cholesterol in the cytofacial leaflet of lysosomes is specifically involved in and essential for activation of mTORC1. Taken together, this study demonstrates that our inducible membrane site-specific cholesterol depletion systems serve as a powerful tool to investigate the site-specific regulatory function of cellular cholesterol.

## Discussion

Depletion of cellular cholesterol by MβCD is one of the most frequently employed methods for cellular lipid manipulation (23). Although the method has long served as a standard protocol for testing the involvement of cholesterol in various cellular processes for lack of alternatives, the method suffers from major shortcomings, including severe deformation of the cell membranes leading to cell death and nonspecific alteration of cell physiology and signaling processes. Inhibition of de novo cholesterol biosynthesis by statins causes similar side effects. They are also slow methods that require long incubation periods. As accumulating evidence demonstrates the involvement of cholesterol in diverse cellular processes (13, 18, 19, 20, 50, 51, 52), a faster and safer alternative to manipulate the cellular cholesterol concentration in a spatiotemporally specific manner has been highly sought after. The method presented in this report satisfactorily meets this need and fulfills the requirements for speed, spatiotemporal specificity, and experimental convenience. Bacterial cholesterol oxidase has been long used for depleting cholesterol in the OPM (31). Cholesterol oxidases from various sources are constitutively active enzymes that act on any cholesterol-containing membranes and thus their exogenous overexpression in mammalian cells could seriously damage cell membranes. To overcome this obstacle that has limited its use as a genetically encoded intracellular agent, Chernov *et al.* (32) recently developed an inducible fluorescent split cholesterol oxidase. This elegant method is based on fragment complementation of split fragments of a green fluorescence protein-cholesterol oxidase fusion protein by FRB-FKBP-based chemically induced dimerization. Although the method successfully addressed the problem of uncontrolled membrane damages due to constitutive activity of cholesterol oxidase and allowed some degree of temporal control, it did not confer spatial control. Also, maturation of the fully active cholesterol oxidase requires several hours, making it difficult to temporally control with this system many cholesterol-mediated processes on physiological time scales. To achieve rapid spatiotemporal control of cholesterol oxidase activity, we took a completely different approach to cholesterol oxidase engineering. Guided by the well-established principles of membrane-protein interactions (35), we engineered *st*CholOx to suppress its membrane binding without affecting its catalytic machinery, which was confirmed by the cholesterol oxidase activity assay and the SPR analysis. One of our mutants, WVR, showed greatly compromised membrane affinity in SPR analysis and caused minimal cholesterol depletion when exogenously expressed in mammalian cells. This allowed us to induce spatially specific cholesterol oxidase targeting and cholesterol depletion by FRB-FKBP-based chemically induced dimerization. That is, FKBP_2_-mTagBFP-WVR was specifically targeted to either Lyn-FRB-localized PM or LAMP1-FRB-decorated lysosomes. Also, spatially specific membrane targeting of FKBP_2_-mTagBFP-WVR and concurrent cholesterol depletion were completed within 10–15 min, which is comparable to the reported time scale of rapamycin (or rapalog) action that is governed by diffusion and cell permeation of the reagent (43, 44). When combined with spatial specificity, this essentially spontaneous response enabled us to control cholesterol depletion in a spatiotemporally specific manner for the first time. It should be noted that although we employed the ratiometric cholesterol quantification method to validate spatiotemporally specific cholesterol depletion, one can also use other semi-quantitative methods, such as membrane localization of a genetically encoded GFP-tagged perfringolysin O-D4 domain derivative (53), to monitor cholesterol depletion, as long as its expression level is carefully controlled to circumvent overexpression artifacts (26).

Two functional studies on cellular cholesterol demonstrate the power and utility of our spatiotemporally specific cholesterol depletion method. Although regulatory roles of PM cholesterol have been extensively reported, a large majority of these studies have treated cholesterol as a main component of lipid rafts (6, 7, 8, 9, 10, 11, 12), without spatial distinction as to whether cholesterol in IPM or OPM is involved in regulation. We thus focused in this study on the Wnt-β-catenin signaling system in which IPM cholesterol has been reported to control the signaling activity by specifically and directly interacting with a scaffolding protein, Dvl, which coordinates the formation of Wnt signalosomes (20, 21, 25). As reported previously (25), stimulation by a canonical Wnt ligand, Wnt3a, raises [Chol]_*i*_, which leads to stabilization of β-catenin. Induced PM targeting of FKBP_2_-mTagBFP-WVR rapidly reduces [Chol]_*i*_ below its basal level without changing [Chol]_*o*_ or [Chol]_*lys*_, which then causes destabilization of β-catenin, leading to termination of Wnt-β-catenin signaling. Furthermore, site-specific depletion of lysosomal cholesterol has no effect on β-catenin stability. These results unambiguously verify the notion that IPM cholesterol plays a direct and specific role in modulating Wnt-β-catenin signaling. They also preclude the possibility that cholesterol in OPM or in other intracellular membranes is directly involved in Wnt-β-catenin signaling.

Likewise, our study clarifies the role of lysosomal cholesterol in regulating mTORC1 activity. Lysosomes play a major role in the regulation of cellular cholesterol homeostasis (54). Thus, processing, transport, and cellular function of lysosomal cholesterol have been extensively studied in the past decade (54). Although much is known about processing of cholesterol ester in lysosomes and shuttling of free cholesterol from the lysosomal lumen to the lysosomal membrane and subsequent export by NPC1 and NPC2 (54), it still remains unknown how cholesterol is distributed between the luminal and cytofacial leaflets of the lysosomal membrane and whether cholesterol in the cytofacial or luminal membrane is important for lysosomal mTORC1 activity (48, 49). An excellent spatiotemporal correlation between [Chol]_*lys*_ and mTORC1 signaling activity as well as the lack of effect of IPM cholesterol depletion on mTORC1 strongly support the notion that cholesterol in the cytofacial leaflet of lysosomes is directly and specifically involved in regulating mTORC1 activity. It should be noted that our ratiometric sensor does not allow for quantification of cholesterol in the luminal leaflet of lysosomes and thus our results cannot preclude the possibility that depletion of cholesterol in the cytofacial leaflet may also lower cholesterol in the luminal leaflet through rapid flip-flop.

In summary, this study establishes a new robust method to deplete cellular cholesterol in a spatiotemporally specific manner, which enables unambiguous determination of the site-specific function of cellular cholesterol. Although the current study focuses on cholesterol at the PM and lysosomes, the same approach can be applied to investigation of cholesterol in any cytofacial membrane of organelles, including mitochondria and the endoplasmic reticulum.

## Data availability

All described data are contained within this article. The raw data will be shared upon request: Contact Wonhwa Cho (University of Illinois at Chicago, Email: wcho@uic.du).

## Conflict of interest

The authors declare that they have no conflicts of interest with the contents of this article.
